# Membrane electrode assembly design to prevent CO_2_ crossover in CO_2_ reduction reaction electrolysis

**DOI:** 10.1038/s42004-022-00806-0

**Published:** 2023-01-03

**Authors:** Hung-Ming Chang, Iryna V. Zenyuk

**Affiliations:** 1 Department of Chemical and Biomolecular Engineering, University of California Irvine, Irvine, CA USA; 2 National Fuel Cell Research Center, University of California Irvine, Irvine, CA USA

**Keywords:** Carbon capture and storage, Electrocatalysis

## Abstract

To reach a net-zero energy economy by 2050, it is critical to develop negative emission technologies, such as CO_2_ reduction electrolyzers, but these devices still suffer from various issues including low utilization of CO_2_ because of its cross-over from the cathode to the anode. This comment highlights the recent innovative design of membrane electrode assembly, utilizing a bipolar membrane and catholyte layer that blocks CO_2_ cross-over and enables high CO_2_ single-pass utilization.

Low temperature (below 100 °C) CO_2_ reduction reaction (CO_2_RR) electrolyzers are promising negative emission technologies that convert CO_2_ to various value-added products^1,2^. These electrolyzers can be easily integrated with renewable power generation (solar and wind) to operate on renewable electricity. For CO_2_RR technologies to be commercially viable they must operate at high: (i) Faradaic efficiency (FE), (ii) conversion rate, (iii) selectivity and (iv) conversion efficiency^3^. It is challenging to achieve all of these four conditions in a single cell experiment, but it is even more difficult to translate the findings to a scaled-up electrolyzer stack system. Specific interest for low temperature electrolysis is its ability to generate multicarbon (C_2+_) products, such as ethylene, ethanol, and propanol because of their commercial value. Recently, significant advances were made to reach competitive product selective current densities (>100 mA cm^−2^) in CO_2_RR to C_2+_ products, with overall good stability. This was achieved with careful catalyst nanoparticles design, their integration within the gas diffusion layers to make gas diffusion electrodes, and with tailoring local environments by using ionomers or liquid electrolytes to achieve neutral or alkaline environments. This alkaline/neutral environment is essential for CO_2_RR cathodes because of the preferential formation of H_2_ over C_2+_ products in an acidic environment. Only very recently^4^ researchers realized that basic chemistry is a big issue for CO_2_RR technology in alkaline environments: The rapid reaction of CO_2_ with OH^-^ in alkaline media to form CO_3_^2−^ is a thermodynamically favored reaction that results in a loss of CO_2_, consumption of OH^−^ and non-steady-state operating conditions, where electrolyte is consumed:

1 $${2{OH}}_{\,\left({aq}\right)}^{\,-}+{{CO}}_{2\left(g\right)}\to {{CO}}_{3\,\left({aq}\right)}^{\,2-}+{H}_{2}{O}_{(l)} \ {\triangle G}^{o}=\,-56\,{kJ}\,{{mol}}^{-1}$$

Interestingly, it requires much larger energy to regenerate CO_2_ and OH^−^ from aqueous CO_3_^2−^, and some studies suggest this value to be >230 kJ mol^−1^ ^5^. Overall, the energy stored in CO_2_RR electrolyzer is around 100–130 kJ/mol of electrons. Therefore, if one needs to regenerate electrolyte after the operation, it will be more energy intensive to do so than the energy stored in C_2+_ products achieved through electrolysis. This is an inherent issue in alkaline/neutral media that many earlier studies have overlooked and only in the course of the last 2 years, or so, studies have started incorporating single-pass utilization (SPU) of CO_2_ as one of the major metrics for the efficiency of the CO_2_RR electrolysis. This metric is defined as the fraction of CO_2_ converted electrochemically to the total input CO_2_:

2 $${SPU}=\frac{{C}_{{electrochem}}}{{C}_{{in}}}$$

where the upper limit of SPU was shown to depend on cross-over CO_2_ (or loss of CO_2_ to electrolyte):

3 $${SPU}{{\_}}\max =\frac{{C}_{{electrochem}}}{{C}_{{electrochem}}+{c}_{{cross}}}$$

The crossover concentration depends on the ability of CO_2_ to react with OH^-^ to form carbonate and bicarbonate products that then cross the membrane and form CO_2_ on the anode side. The SPUmax values are currently <25% for C_2+_ products, resulting in overall net negative energy balance of the electrolyzer operation.

Recently, Sinton, Sargent and collaborators^6^ have reported a novel electrolyzer design based on a bipolar membrane (BPM) that aims to block CO_2_ crossover to the anode side, achieving a remarkable CO_2_ SPU of 78%, a factor of 10× improvement compared to earlier works. The innovative design they propose is based on the requirements that (i) CO_2_ must not cross to the anode through the alkaline exchange membrane (AEM) and (ii) CO_2_ that is reacted to form carbonates and bicarbonates on the cathodes side must be regenerated internally within the electrolyzer. The first requirement was resolved by incorporation of a BPM, but not in zero-gap configuration, where a cation exchange membrane (CEM) will face the cathode and reduce the FE of the CO_2_RR. The CEM side of the BPM is in contact with a non-buffer stationary catholyte (SC) layer, as shown in Fig. 1a. By allowing this SC-layer, the CEM is sufficiently removed from the cathode catalyst layer to not induce an acidic environment in the catalyst layer. Key to this design that addresses the second requirement is a non-flowing and non-buffer SC-layer, as a flowing catholyte layer would remove CO_2_ that was converted to carbonate/bicarbonate, resulting in low SPU. The authors have shown (Fig. 1a) that carbonate and bicarbonate ions recombine with protons to regenerate CO_2_ and that the regenerated CO_2_ diffuses back to the copper catalyst layer and reacts there. By optimizing feed-in CO_2_ concentrations and the thickness of the non-buffer SC-layer to maximize CO_2_ regeneration and minimize diffusion losses, while maintaining a high pH at the cathode catalyst layer, one can achieve unprecedented SPU. The team has significant expertize in the design of BPMs and the results suggest that the electrolyzer with the SC BPM membrane electrode assembly at 200 mA cm^−2^ has similar cell voltage compared to an AEM electrolyzer but with significantly higher SPU (78%). Furthermore, they have shown >50 h stability operating at 200 mA cm^−2^ with restricted CO_2_ inlet, being a competitive stability result to the published data.

**Fig. 1:**
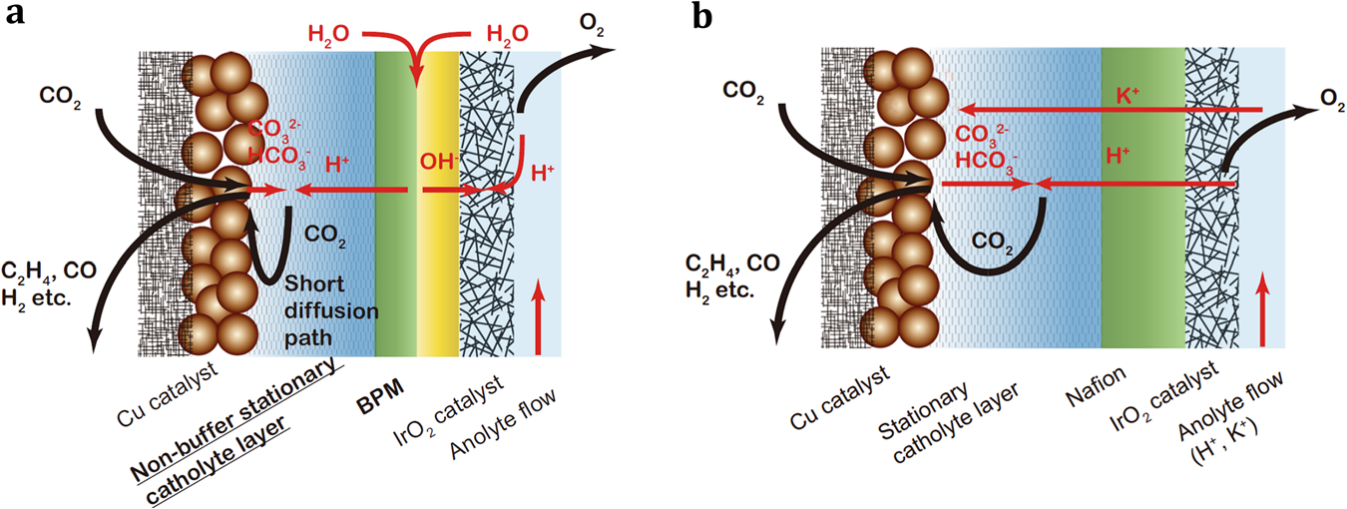
A schematic of the CO_2_RR electrolyzer and relevant transport processes. The CO_2_RR electrolyzer utilizing (**a**) a bipolar membrane (BPM) and (**b**) a cation exchange membrane (CEM). Copyright: Modified from ref. ^6^., used under CC BY.

In the same work, Sinton, Sargent and collaborators^6^ have further extended this innovative CO_2_RR electrolyzer concept to a CEM electrolyzer, as shown in Fig. 1b. The advantage of CEM over BPM is that it eliminates the water dissociation reaction at the bipolar junction and reduces the overall thickness of the membrane. They have shown that the CO_2_ crossover with the CEM concept was eliminated too. The CEM electrolyzer had an overall lower cell voltage and comparable SPU. The necessity of the anolyte flow is to provide cations that are needed to shield negative charge in the cathode catalyst layer, the so-called “cation effect”^7^, to enable high FE toward C_2+_ products over the hydrogen evolution reaction. As the authors pointed out, without acid and salt addition the steady-state cannot be reached as the pH gradients will be eliminated with co-ion transport and neutralization.

## Outlook

The work by Sinton, Sargent and collaborators^6^ has opened a new research area in methods to improve SPU of CO_2_RR electrolyzers by enabling CO_2_ regeneration through smart electrolyzer design. The authors suggest area of improvements including use of ionic liquids in the catholyte and tuning the morphology (porosity, pore sizes, tortuosity and wettability) of the SC-layer, which is currently stacked PVDF layers. In terms of larger scale fabrication, SC-layer pretreatment and degassing need to be developed for roll-to-roll fabrication processes. Several issues that might arise include potassium carbonate precipitation, resulting in challenges to long-stability operation. The cation effect might still be important for the system under study and understanding the non-buffering electrolyte selection with various cations can be important.

Although CO_2_ SPU has been apparently improved, the cell voltage still has room for improvement. Recent work by Vass at el^8^. suggested coupling CO_2_RR with value-added anode products or with reactions that have lower overpotential on the anode side, enhancing the energy efficiency. As the electrolyzers operate at higher current densities, the challenge of losing liquid products through a BPM will also need to be addressed.

In an alternative approach one can use CEM and local acidic environments to prevent CO_2_ reaction with OH^-^. However, in acidic environments, hydrogen evolution reaction (HER) is kinetically favorable compared to CO_2_RR because adsorbed H competes with the adsorption of CO. Wang, Sargent and collaborators^9^ have used density functional theory to show that addition of Pd to Cu can help local CO binding and promote C-C coupling, weakening the H-binding energy and therefore suppressing HER. This alternative approach achieved a CO_2_ to C_2+_ SPU of 60% at 500 mA cm^−2^ and will serve as a new direction to suppress CO_2_ crossover.

A third direction is to modify the anode stream to enable efficient CO_2_ separation, as for an AEM a typical mixture is 20–40% O_2_ with 60–80% CO_2_, which cannot be fed directly into the cathode, as the oxygen reduction reaction would dominate over the CO_2_RR. Recent work by Sinton, Sargent and collaborators^10^ reported an all liquid anode to enable easy separation of gaseous CO_2_ and liquid products from the anode reactions. They have used organic oxidation reaction (OOR) on the anode side, specifically glucose oxidation reaction, showing high SPU of 48% and lower cell voltage compared to OER. Other OOR could be explored to enable CO_2_ recovery and also to generate value-added products on the anode.
